# Pro-Arrhythmic Ventricular Remodeling Is Associated With Increased Respiratory and Low-Frequency Oscillations of Monophasic Action Potential Duration in the Chronic Atrioventricular Block Dog Model

**DOI:** 10.3389/fphys.2019.01095

**Published:** 2019-08-23

**Authors:** David Jaap Sprenkeler, Jet D. M. Beekman, Alexandre Bossu, Albert Dunnink, Marc A. Vos

**Affiliations:** Department of Medical Physiology, Division of Heart and Lungs, University Medical Center Utrecht, Utrecht, Netherlands

**Keywords:** chronic AV block dog, electrical remodeling, respiration, action potential duration, low-frequency oscillations

## Abstract

In addition to beat-to-beat fluctuations, action potential duration (APD) oscillates at (1) a respiratory frequency and (2) a low frequency (LF) (<0.1 Hz), probably caused by bursts of sympathetic nervous system discharge. This study investigates whether ventricular remodeling in the chronic AV block (CAVB) dog alters these oscillations of APD and whether this has consequences for arrhythmogenesis. We performed a retrospective analysis of 39 dog experiments in sinus rhythm (SR), acute AV block (AAVB), and after 2 weeks of chronic AV block. Spectral analysis of left ventricular monophasic action potential duration (LV MAPD) was done to quantify respiratory frequency (RF) power and LF power. Dofetilide (0.025 mg/kg in 5 min) was infused to test for inducibility of Torsade de Pointes (TdP) arrhythmias. RF power was significantly increased at CAVB compared to AAVB and SR (log[RF] of −1.13 ± 1.62 at CAVB vs. log[RF] of −2.82 ± 1.24 and −3.29 ± 1.29 at SR and AAVB, respectively, *p* < 0.001). LF power was already significantly increased at AAVB and increased even further at CAVB (−3.91 ± 0.70 at SR vs. −2.52 ± 0.85 at AAVB and −1.14 ± 1.62 at CAVB, *p* < 0.001). In addition, LF power was significantly larger in inducible CAVB dogs (log[LF] −0.6 ± 1.54 in inducible dogs vs. −2.56 ± 0.43 in non-inducible dogs, *p* < 0.001). In conclusion, ventricular remodeling in the CAVB dog results in augmentation of respiratory and low-frequency (LF) oscillations of LV MAPD. Furthermore, TdP-inducible CAVB dogs show increased LF power.

## Introduction

Repolarization lability, quantified as beat-to-beat fluctuations in action potential duration (APD), is known to contribute to arrhythmogenesis (Thomsen et al., 2004, 2007). An increased beat-to-beat repolarization variability has been found in patients with a high risk of ventricular arrhythmias, such as patients with heart failure (Berger et al., 1997; Hinterseer et al., 2010), ischemia (Murabayashi et al., 2002), long QT-syndrome (Hinterseer et al., 2008, 2009), hypertrophic cardiomyopathy (Atiga et al., 2000), or hypertension with left ventricular hypertrophy (Piccirillo et al., 2002). In these patients, adverse cardiac remodeling has led to heterogeneous downregulation of repolarizing ionic currents and a disruption of normal Ca^2+^ handling (Armoundas et al., 2001). As a result, the so called “repolarization reserve” is reduced, making the process of repolarization unstable and prone to arrhythmogenic challenges (Roden, 1998).

In addition to beat-to-beat variations in repolarization, the APD also oscillates at a broader range of frequencies. First, APD fluctuates with respiration, which appears to be independent of the respiratory effects on heart rate (Hanson et al., 2012). Second, APD oscillates at a LF of around 0.1 Hz, which has been attributed to LF bursts of sympathetic nerve terminals on the ventricular myocardium (Hanson et al., 2014). While a sympathetically mediated LF pattern of arterial blood pressure (known as Mayer waves) is well-known (Malpas, 2002), oscillations at 0.1 Hz have only recently been found in APD as well (Hanson et al., 2014; Porter et al., 2018). Moreover, these fluctuations have also been identified on the surface ECG as changes in T wave vector angle between consecutive beats, referred to as “periodic repolarization dynamics” (PRD; Rizas et al., 2014).

However, it is unknown whether APD oscillations at these frequency bands (i.e., respiratory and LF) reflect normal physiology or whether they are linked to the occurrence of ventricular arrhythmias. In this regard, a computational modeling study showed that during Ca^2+^ overload and reduction of repolarizing currents, APD oscillations could become arrhythmogenic and elicit afterdepolarizations (Pueyo et al., 2016). Furthermore, in clinical studies of post-myocardial infarction patients, PRD appears to be a strong independent predictor of all-cause mortality (Hamm et al., 2017; Rizas et al., 2017). Therefore, we could hypothesize that these oscillations are altered by ventricular remodeling, thereby further destabilizing repolarization and contributing to arrhythmogenesis.

In the present study, we evaluated both respiratory and low-frequency (LF) oscillations of APD in the chronic complete AV block dog model. In this arrhythmogenic animal model, creation of complete AV block results in cardiac remodeling and reduction of repolarization reserve. Administration of anesthesia and a pro-arrhythmic drug, i.e., the *I*_Kr_ blocker dofetilide, will act as the final “hit” on repolarization, resulting in electrical storm with multiple episodes of Torsades de Pointes arrhythmias (TdP) in approximately 75% of the dogs (Oros et al., 2008). This model has been widely used in our laboratory and by others to investigate the mechanisms of arrhythmogenesis in the remodeled heart (Thomsen et al., 2007; Zhou et al., 2008; Oosterhoff et al., 2010; Dunnink et al., 2012). Therefore, we could use this model to investigate whether ventricular remodeling alters respiratory and LF oscillations of APD.

The current study is a retrospective analysis of previously performed experiments in which we analyzed respiratory and LF oscillations under different conditions of remodeling, i.e., during sinus rhythm (SR), acutely after creation of AV block (AAVB) and after (at least 2 weeks) of remodeling at chronic AV block (CAVB). In addition, we compared inducible with non-inducible CAVB dogs, to evaluate the relevance of these oscillations for arrhythmogenesis.

## Materials and Methods

Animal handling was in accordance with the “Directive 2010/63/EU of the European Parliament and of the Council of 22 September 2010 on the protection of animals used for scientific purposes” and the Dutch law, laid down in the Experiments on Animals Act. All experiments were performed with approval of the Central Authority for Scientific Procedures on Animals (CCD).

We did a retrospective analysis on electrophysiological data in our database of dog experiments executed between 2014 and 2017, which were done to study the mechanisms of TdP arrhythmias or to test new anti-arrhythmic agents or interventions. In order to maintain a homogenous population, only dogs remodeled on their own idioventricular rhythm (IVR) were included, thereby excluding dogs that were chronically paced from the right ventricular apex (RVA), which has shown to influence the remodeling process. Furthermore, only baseline recordings before the administration of any anti-arrhythmic drugs were used for the analysis to exclude the effect of these interventions on the oscillatory pattern of APD. In addition, we excluded experiments that had a baseline recording shorter than 5 min or recordings that had too much ectopy or noise (approximately more than 10% of the recording).

### Animal Experiments

Detailed description of the experimental set-up has been reported previously (Dunnink et al., 2010, 2012). In brief, all experiments were performed under general anesthesia with induction *via* pentobarbital sodium 25 mg/kg i.v. and maintained by isoflurane 1.5% in O_2_ and N_2_O, 1:2. Animals were ventilated with positive pressure ventilation at a rate of 12 breaths/min. Next, monophasic action potential catheters (Hugo Sachs Elektronik, March, Germany) were introduced *via* the femoral artery and vein into the heart to measure the left ventricular and right ventricular monophasic action potential duration (LV and RV MAPD). In the initial experiment, complete atrioventricular (AV) block was created by radiofrequency ablation of the proximal His bundle. Subsequently, the dogs remodeled for at least 2 up to 5 weeks on IVR.

In all experiments, after a baseline measurement of at least 5 min, inducibility of TdP arrhythmias was tested by infusing the *I*_Kr_ blocker dofetilide (0.025 mg/kg in 5 min or before the first TdP). TdP was defined as a run of five or more short-coupled (occurring before the end of the T wave) ectopic beats, with polymorphic twisting of the QRS-axis. When ≥3 TdP arrhythmias occurred in the first 10 min after the start of infusion, the dog was considered inducible. During baseline and dofetilide challenge, all subjects were paced from the RV-apex at 60 beats per minute.

### Data Analysis

For this retrospective analysis, we used LV MAPD recordings at SR, AAVB, and CAVB conditions. The monophasic action potential was recorded with EP Tracer (Cardiotek, Maastricht, The Netherlands) at a sampling frequency of 1,000 Hz. LV MAPD was measured offline semi-automatically from the initial peak to 80% of repolarization using custom-made software in MATLAB (MathWorks, Natick, USA). In addition, for analysis of LF oscillations, the absolute difference in LV MAPD between two consecutive beats was calculated. Any extrasystolic beats and the subsequent post-extrasystolic beats were removed. The 5-min time series of MAPD or MAPD difference was detrended and interpolated at 4 Hz *via* cubic spline interpolation to get evenly spaced samples. Data series were split into epochs of 512 samples with 50% overlap. Spectral analysis was performed in MATLAB with Welch’s periodogram and a Hanning window to derive the power spectral density (PSD). The power of the frequency bands was calculated by integrating the area under the PSD plot for bandwidths of different frequencies. For the respiratory frequency (RF), we selected a frequency band between 0.19 and 0.21 Hz, since all dogs were ventilated at 12 breaths per minute (every 5 s, 0.2 Hz). For the LF oscillations, we used a frequency band between 0.04 and 0.15 Hz as has been used in previous studies (Hanson et al., 2014; Porter et al., 2018), since the frequency of sympathetic bursts can differ between individual subjects.

Measurement of RR-interval and QT-interval was performed in lead II of the surface ECG. QT-interval was corrected for heart rate (QT_c_) with the van der Water formula (Van de Water et al., 1989). Short-term variability (STV) of LV MAPD was calculated over 31 consecutive beats using the formula: $\mathrm{STV}=\sum \left|D_{n+1}-D_{n}\right|/30\times \sqrt{2}$, where *D* represents LV MAPD.

### Statistical Analysis

Numerical values are expressed as mean ± standard deviation (SD). Logarithmic transformation of both RF and LF was used to correct for skewness of the data. Normality of the transformed data was checked with the Shapiro-Wilk test. Group comparison was done with an unpaired Students *t*-test. Group comparison of more than two groups was performed with a one-way analysis of variance (ANOVA) with Tukey’s correction for multiple comparisons. *p* equal to or smaller than 0.05 was considered significant. GraphPad Prism 6 (GraphPad Software, Inc., La Jolla, CA, USA) was used for the statistical analysis.

## Results

A total of 39 experiments in 29 adult mongrel dogs (13 males, 16 females, weight 25 ± 2.5 kg) were used for the analysis. We included 10 dogs in SR, 10 dogs in AAVB, and 19 dogs in CAVB (14 inducible, 5 non-inducible). Of three dogs, data of both AAVB and CAVB experiment were used.

### Baseline Electrophysiological Parameters

Baseline electrophysiological data at the three conditions (SR, AAVB, and CAVB) are depicted in Table 1. As expected, QT-interval increased acutely after the creation of AV block, due to the sudden drop in heart rate. In CAVB, electrical remodeling has occurred as seen by a significant increase in QT, QTc, and LV MAPD. Furthermore, STV is significantly increased, reflecting a reduced repolarization reserve. Table 2 shows electrophysiological parameters separately for the non-inducible and inducible CAVB dogs. Only STV appears to be higher in the inducible dogs; however, this did not reach statistical significance (*p* = 0.08).

**Table 1:** Baseline electrophysiological parameters.

	SR (*n* = 10)	AAVB (*n* = 10)	CAVB (*n* = 19)
RR (ms)	557 ± 32	1,000^*^	1,000
QT (ms)	267 ± 15	357 ± 19^*^	407 ± 56^§^
QTc (ms)	305 ± 15	357 ± 19	407 ± 56^§^
LV MAPD_80_ (ms)	200 ± 11	243 ± 14^*^	275 ± 36^§^
STV LV MAPD_80_ (ms)	0.31 ± 0.06	0.54 ± 0.30	1.20 ± 0.80^§^

* *p < 0.05 vs. SR*.

§ *p < 0.05 vs. AAVB*.

**Table 2:** Baseline electrophysiological parameters of inducible and non-inducible dogs.

	Inducible (*n* = 14)	Non-inducible (*n* = 5)
RR (ms)	1,000	1,000
QT (ms)	414 ± 60	388 ± 43
QTc (ms)	414 ± 60	388 ± 43
LV MAPD_80_ (ms)	283 ± 34	260 ± 34
STV LV MAPD_80_ (ms)	1.39 ± 0.83	0.64 ± 0.40

### Respiratory Oscillations

**Figure 1:**
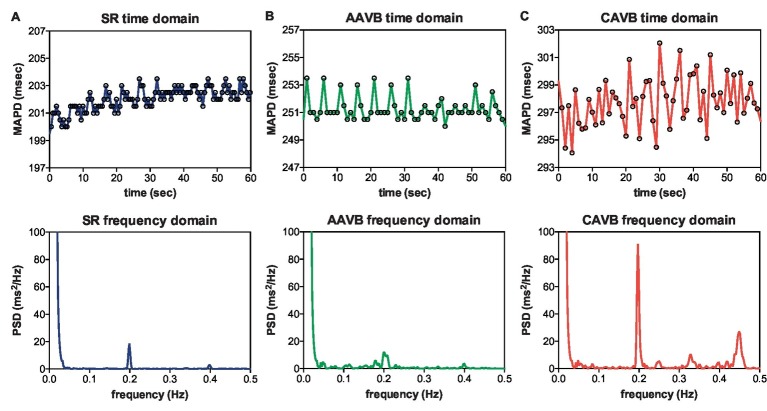
Respiratory frequency oscillations in time and frequency domain. Representative examples of oscillations in monophasic action potential duration (MAPD) in the time domain (top) and frequency domain (bottom) during **(A)** sinus rhythm (SR), **(B)** acutely after creation of AV block (AAVB), and **(C)** after remodeling at chronic AV block (CAVB). A clear increase in a 0.2 Hz oscillation is seen at CAVB.

**Figure 2:**
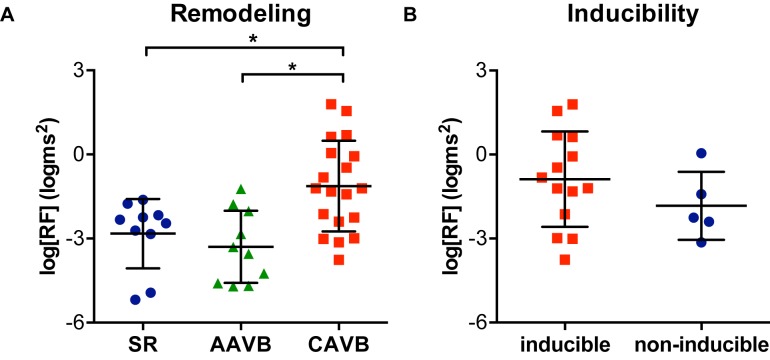
Respiratory oscillations of monophasic action potential duration. **(A)** The logarithmic transformed power of respiratory oscillations of APD (log[RF]) at sinus rhythm (SR), acutely after AV block (AAVB), and at chronic AV block (CAVB). **(B)** log[RF] of the inducible vs. the non-inducible CAVB dogs. ^*^p < 0.05.

Figure 1 shows an example of the respiratory fluctuations in MAPD of dogs in SR, AAVB, and CAVB in both, time domain and frequency domain. At SR and AAVB, low amplitude respiratory oscillations of LV MAPD were present, while at CAVB, larger oscillations are seen around the respiratory frequency. Figure 2 displays the quantified logarithmic RF power (log[RF]) of the analyzed dogs. The remodeling process (Figure 2A) resulted in augmentation of the variability at the respiratory frequency, as seen by a significant increase in a log[RF] of −2.55 ± 1.48 and −2.99 ± 1.20 at SR and AAVB, respectively, to a log[RF] of −0.82 ± 1.53 (*p* < 0.001) at CAVB. When comparing inducible with non-inducible dogs, no significant difference could be found in RF power (Figure 2B).

### Low-Frequency Oscillations

**Figure 3:**
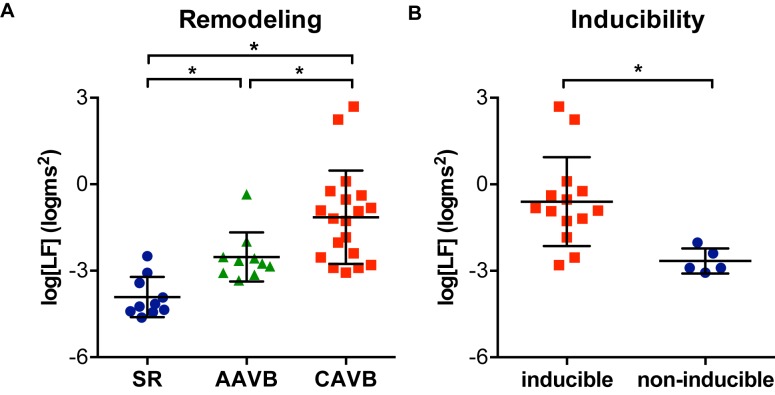
Low-frequency (LF) oscillations of monophasic action potential duration. **(A)** The logarithmic transformed power of LF oscillations of APD (log[LF]) at sinus rhythm (SR), acutely after AV block (AAVB), and at chronic AV block (CAVB). **(B)** log[LF] of the inducible vs. the non-inducible CAVB dogs. ^*^p < 0.05.

**Figure 4:**
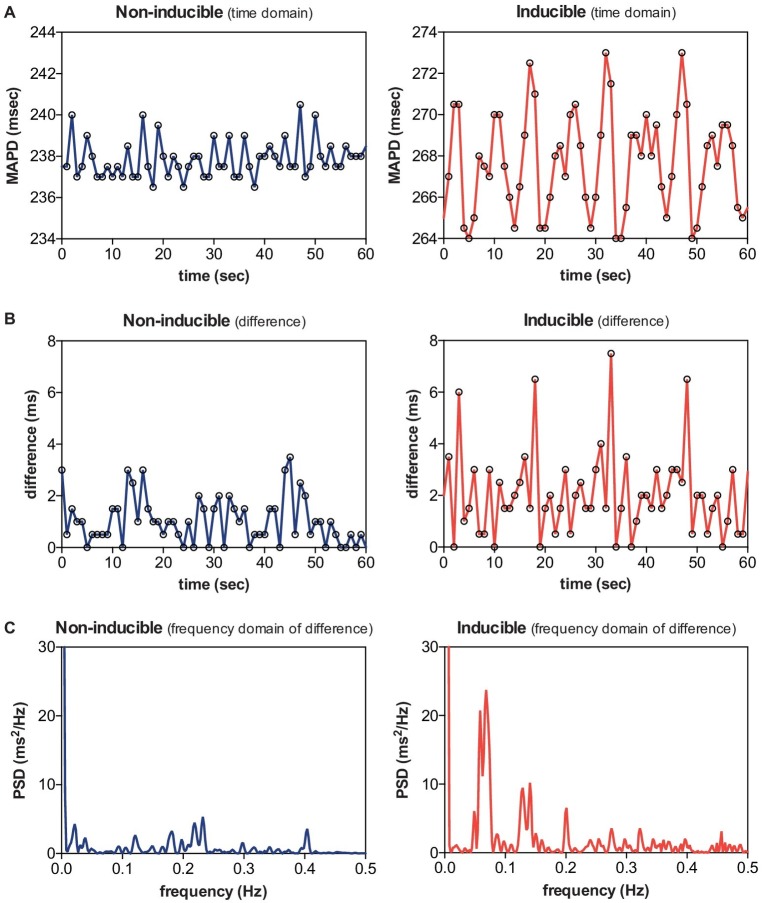
Low-frequency (LF) oscillations in a non-inducible dog vs. an inducible dog. A representative example of MAPD **(A)**, MAPD difference in the time domain **(B)**, and MAPD difference in the frequency domain **(C)** of a non-inducible dog (left) and an inducible dog (right). A clear LF pattern in MAPD difference can be discerned in the inducible dog.

Next, we examined LF oscillations in MAPD difference in SR, AAVB, and CAVB. As depicted in Figure 3A, already a significant rise in LF power can be seen at AAVB compared to SR, which further increased after 2 weeks of remodeling (log[LF] of −3.91 ± 0.70 at SR, vs. −2.52 ± 0.85 at AAVB, and −1.14 ± 1.62 at CAVB, *p* < 0.001). Finally, we looked for differences of these oscillations between inducible and non-inducible CAVB dogs. A representative example of the MAPD during the 5-min recording of an inducible and a non-inducible dog is shown in Figure 4A. A clear oscillation can be observed in the inducible subject, with a rhythmic fluctuation in MAPD. This oscillatory behavior of MAPD can more clearly be discerned when the difference between consecutive beats is plotted against time (Figure 4B): approximately every 10–15 s, a clear increase in the variability between successive beats is seen. When this MAPD variability is visualized in the frequency domain by spectral analysis (Figure 4C), a prominent peak appears in the LF band (0.04–0.15 Hz). As depicted in Figure 3B, the inducible dogs demonstrated a significant higher LF power of MAPD difference when compared to non-inducible dogs (log[LF] −0.6 ± 1.54 vs. −2.56 ± 0.43, *p* < 0.001).

## Discussion

In this retrospective analysis of previously performed animal experiments, we demonstrated that (1) respiratory frequency oscillations of MAPD are increased after electrical remodeling, but they do not differ between inducible and non-inducible dogs and (2) LF oscillations of MAPD difference are already increased at AAVB and rise even further at CAVB. Furthermore, these 0.1 Hz oscillations are more pronounced in CAVB dogs that are susceptible to dofetilide-induced TdP arrhythmias.

### The Chronic Atrioventricular Block Dog Model to Study the Effects of Electrical Remodeling on Arrhythmogenesis

A variety of structural heart diseases (e.g., myocardial infarction, pressure overload due to hypertension or aortic stenosis, volume overload as seen in valvular regurgitation) can lead to pathological cardiac remodeling, causing downregulation of potassium currents (*I*_to_, *I*_Ks_, *I*_Kr_, and *I*_K1_) (Long et al., 2015), enhanced late Na^+^-current (*I*_Na-L_) (Antzelevitch et al., 2014), and Ca^2+^ handling abnormalities (Sipido et al., 2002). As a result, repolarization reserve is reduced, making the heart prone to repolarization-dependent ventricular arrhythmias. The CAVB dog model, as used in this study, is a model of ventricular remodeling and reduced repolarization reserve that reflects the vulnerable patient at risk for these arrhythmias. In this model, it has been shown that beat-to-beat variability of APD, quantified as STV, is a better marker of reduced repolarization reserve and pro-arrhythmia than APD prolongation itself (Thomsen et al., 2004). STV is significantly increased at CAVB compared to AAVB and dogs susceptible to dofetilide-induced TdP arrhythmias show a further rise in STV prior to occurrence of arrhythmias (Thomsen et al., 2007).

In the current study, we have shown that not only successive beat-to-beat fluctuations of APD exists in the CAVB dog, but also that the APD oscillates at other frequency bands. This is in line with previous studies that have demonstrated important contributions of variation in heart rate (Hnatkova et al., 2013), respiration (Hanson et al., 2012), and autonomic nervous system activity (Baumert et al., 2011) on APD variability. Concerning heart rate, a complex and dynamic APD to heart rate relation exists that is highly individual-specific and contains significant hysteresis effects (Malik et al., 2008). In this study, we have eliminated important heart rate effects on APD by including only dogs that were paced during the experiments. Therefore, we could focus solely on the respiratory and autonomic influences on APD.

### Respiratory Oscillations of Action Potential Duration in the Chronic Atrioventricular Block Dog

While heart rate is well-known to fluctuate with respiration, it was recently shown by Hanson et al. that APD, measured as activation recovery interval (ARI) from the intracardiac electrogram, also displays rhythmic fluctuations in synchrony with respiration, even when heart rate was controlled by pacing. The authors suggested multiple mechanisms for the respiratory oscillations of APD. One of these, mechano-electrical feedback, relates to the modulation of electrophysiology by changes in ventricular loading conditions. Both in animal models as in patient studies, a direct effect of altered mechanical load on APD have been found; increased ventricular load resulted in shortening of the APD, while reduction in load was associated with prolongation of the APD (Levine et al., 1988; Zabel et al., 1996). Stretch-activated ion-channels or alterations in Ca^2+^ handling have been suggested as the underlying molecular mechanism of load-dependent APD changes (Eckardt et al., 2001). Stretch-activated ion channels are non-specific cation (Na^+^, K^+^, and Ca^2+^) channels that open in respond to changes in stretch instead of voltage (Zeng et al., 2000). In addition, mechanical stretch increases Ca^2+^ release from the sarcoplasmic reticulum, which can alter action potential duration *via* negative feedback on the L-type Ca^2+^-channel or by exchange of Ca^2+^ for Na^+^ *via* the Na^2+^-Ca^2+^-exchanger (Iribe et al., 2009). One important physiological mechanism that can alter ventricular loading conditions is the change in intrathoracic pressure difference during respiration. During spontaneous inspiration, intrathoracic pressure drops, causing an increased systemic venous return to the RV, which will shift the interventricular septum into the LV. As a result, left ventricular end-diastolic volume and left ventricular preload will decrease. The opposite will occur during positive pressure ventilation: in that situation, an increase in left ventricular preload will be seen during inspiration (Mitchell et al., 2005). Nevertheless, in either case, a respiratory oscillatory behavior of ventricular loading is present, which could therefore alter APD in a cyclical pattern.

In the present study, we showed that the modulating effect of respiration on APD is enhanced after cardiac remodeling. A possible explanation could be that alternating changes in ventricular loading have greater impact on repolarization, when repolarization reserve is already reduced. This is consistent with a study by Stams et al. in which the effect of preload changes on beat-to-beat variability of APD was studied in the CAVB dog (Stams et al., 2016). The authors used a pacing protocol with either a constant or alternating PQ interval to artificially control preload conditions. They observed that in AAVB, alternating preload had no effect on APD or STV. In contrast, in CAVB dogs pacing with an alternating PQ interval resulted in APD variability and a significantly higher STV compared to conditions of constant preload. Furthermore, blockade of stretch-activated ion current (*I*_SAC_) by streptomycine prevented the increase of STV_LV MAPD_ during alternating preload. Although streptomycine is not a selective (*I*_SAC_)-blocker and has affinity for other ion channels that could affect STV (like L-type Ca^2+^ channels), these results suggest that mechano-electrical feedback *via* specialized stretch-activated ion channels could have profound influence on repolarization during reduced repolarization reserve. This is further supported by a study of Kamkin et al., which showed that isolated cardiomyocytes from hypertrophied ventricles were more sensitive to stretch than control cardiomyocytes, resulting in prolongation of APD at smaller mechanical stimuli (Kamkin et al., 2000). Thus, we may hypothesize that after remodeling and downregulation of repolarizing K^+^-currents, the relative contribution of *I*_SAC_ to the repolarization process is increased; therefore, we observed an augmentation of APD variability caused by changes in respiration-mediated loading conditions.

Interestingly, we did not find a difference in respiratory oscillations between inducible and non-inducible CAVB dogs. In both groups, electrical remodeling reduced repolarization reserve, as we observed by enhanced respiratory fluctuation of APD. A similar finding was reported by the study of Stams et al.: alternating preload, which led to an increase in APD variability, did not result in more TdP arrhythmias compared to conditions of constant preload. Thus, we could assume that an additional trigger is required to create the optimal environment for dofetilide-induced TdP arrhythmias.

### Low-Frequency Oscillations of Action Potential Duration Difference in the Chronic Atrioventricular Block Dog

LF oscillations in the range from 0.04 to 0.15 Hz that are unrelated to respiration have long been observed in both heart rate and arterial blood pressure, and are referred to as Mayer waves (Julien, 2006). These oscillations have been linked to rhythmic bursts of sympathetic nervous system activity; however, the precise mechanism remains controversial. Two theories exist: (1) these oscillations are the effect of a central autonomous oscillator within the central nervous system that fires at a certain frequency and (2) they are the result of a time delay in the baroreflex loop, causing resonance in the feedback system (Malpas, 2002). Either way, states of increased sympathetic activation, such as during tilt test or when blood pressure was artificially lowered, resulted in an increase in the magnitude of Mayer waves (Pagani et al., 1997; Furlan et al., 2000). In addition, blockade of sympathetic drive resulted in a reduction of LF components of both RR interval and blood pressure (Pagani et al., 1997).

In addition to respiratory fluctuations, Hanson et al. also showed that APD displays an oscillatory pattern at Mayer wave frequency (Hanson et al., 2014). Moreover, these LF oscillations increased during autonomic challenge with Valsalva maneuver (Porter et al., 2018). A similar LF oscillatory pattern was found by Rizas et al. in T wave vector changes on the surface-ECG, called periodic repolarization dynamics (PRD; Rizas et al., 2014). These variations in T wave vector could also be increased with exercise and reduced by β-adrenergic blockade, suggesting a role for sympathetic input on the myocardium in the pathogenesis of these oscillations. Furthermore, increased PRD appeared to be a strong predictor of all-cause mortality in a cohort of more than 900 post-MI patients. Combined with a marker of vagal activity (i.e. deceleration capacity), PRD was able to accurately stratify mortality risk in these patients (Hamm et al., 2017). Nevertheless, the cause of death, whether arrhythmic or due to pump failure, was not further specified.

In this regard, the findings of the current study could be of great interest. In this study, we evaluated the effect of ventricular remodeling on LF oscillations, but, more importantly, whether these oscillations were different in dogs susceptible to arrhythmias. For this analysis, the MAPD difference between two consecutive beats was used, instead of MAPD itself. The reason for this is that in the previous study by Rizas et al., PRD was also measured on differences in T wave vector between beats. They observed that approximately every 10 s, the T wave vector changed markedly, while in the intermittent periods T wave vector remained relatively stable. We hypothesized that 0.1 Hz bursts of sympathetic discharge could also result in sudden changes in MAPD, which are more clearly visualized when spectral analysis is done of MAPD difference instead of MAPD.

Consequently, we could show that acutely after creation of AV block, LF power of APD difference is already significantly increased. The sudden drop in cardiac output and blood pressure that occur after creation of AV block will be sensed by baroreceptors in the aortic arch and carotid sinus, which will increase efferent sympathetic input on the heart, while simultaneously reducing parasympathetic firing. We observed this baroreflex-mediated increase in sympathetic tone by augmentation of LF APD oscillations acutely after AV block. More importantly, cardiac remodeling further increased the LF oscillatory behavior of APD difference, predominantly in CAVB dogs susceptible to drug-induced TdP arrhythmias. From the existing literature, it becomes clear that increased sympathetic nervous system activity is an important contributor to repolarization variability and arrhythmogenesis. A study by Johnson et al. in isolated cardiomyocytes showed that the addition of β-adrenergic stimulation to a state of reduced repolarization reserve (by blockade of *I*_Ks_) led to a dramatically increase in beat-to-beat variability of repolarization (BVR; Johnson et al., 2010). In addition, sympathetic stimulation promoted Ca^2+^ overload, spontaneous Ca^2+^-release and the formation of early and delayed afterdepolarizations (EADs/DADs; Johnson et al., 2013). Gallagher et al. found similar results in an *in vivo* canine model of drug-induced LQTS-1. Infusion of HMR1556, an *I*_Ks_ blocker, in combination with isoproterenol resulted in paradoxically increased APD, increased spatial and temporal dispersion of repolarization and reproducible TdP arrhythmias (Gallacher et al., 2007). The same was found in a study of Ter Bekke et al., who used direct left stellate ganglion stimulation instead of pharmacological adrenergic stimulation, combined with Iks blockade (Ter Bekke et al., 2019). A simulation study by Pueyo et al. evaluated the effect of phasic β-adrenergic stimulation on APD dynamics (Pueyo et al., 2016). They observed a LF oscillatory pattern of APD, whose magnitude increased with higher β-adrenergic strength. Interestingly, simulated pathological conditions of Ca^2+^-overload and reduced repolarization reserve (comparable to the CAVB dog model) enhanced the APD oscillations caused by adrenergic stimulation. Therefore, the authors suggested an important role of these oscillations in arrhythmogenesis. In the present study, we could confirm these *in silico* results experimentally in an arrhythmogenic *in vivo* model.

The reason for the clear difference in LF oscillations of APD difference between inducible and non-inducible dogs remains speculative. Two mechanisms can be proposed: either repolarization reserve is even more reduced in the inducible dogs, therefore making the effect of β-adrenergic stimulation on repolarization more prominent and repolarization more vulnerable to arrhythmogenic challenges, or sympathetic output itself (either systematically or due to increased local density of sympathetic neurons) is further enhanced in the inducible dogs, causing increased repolarization instability, Ca^2+^ overload, and triggered activity. Concerning the latter, studies in dogs with chronic AV block and MI have shown that in addition to electrical remodeling, also neural remodeling takes place, as seen by denervation, hyperinnervation, and nerve sprouting (Cao et al., 2000a,b). Regional hyperinnervation, where some regions are more densely innervated than others, combined with heterogeneous electrical remodeling, further enhances spatial dispersion of repolarization; thereby, facilitating the initiation and perpetuation of ventricular arrhythmias.

### Implications

In addition to beat-to-beat variation in APD or QT-interval, we have shown that fluctuations in other frequency bands are altered in subjects with pro-arrhythmic ventricular remodeling and an increased risk of ventricular arrhythmias. Therefore, these oscillations might eventually be used in risk stratification of patients at high risk of sudden cardiac death, who might benefit from implantation of an implantable cardioverter-defibrillator (ICD). While in the current study, MAP-catheters were used, Hanson et al. showed that respiratory and LF oscillations are also measurable on the ARI of intracardiac EGM, which could be obtained from implantable devices. Furthermore, PRD is a non-invasive parameter that can be measured from a converted 12-lead surface ECG, which would make it more suitable for risk stratification prior to ICD implantation. In this regard, PRD is currently being studied as a predictive marker in the multicentre, observational EU-CERT-ICD study (NCT02064192), which evaluates new risk stratification methods that could identify subgroups of patients with low or high risk of ICD-shocks or mortality.

### Study Limitations

Since the CAVB dog is a specific model of ventricular remodeling caused by volume overload, extrapolation of these results to patients with other causes of remodeling (ischemia, infarction, and pressure overload) should be done with caution. Second, an important limitation of the study is its retrospective nature, which made it impossible to control for all, possible confounding, variables. By selecting only dogs remodeled on IVR without control of activation pattern, we tried to keep the analyzed group of dogs as homogeneous as possible. Third, all dogs were mechanically ventilated with positive pressures, which has an opposite effect on loading conditions of the heart compared to spontaneous breathing. Nevertheless, both ventilation techniques result in an oscillatory pattern, albeit with a shift in phase. Next, all experiments were done under general anesthesia, which has profound effects on the autonomic nervous system. In addition, no direct measurements of neural activity were done to confirm that the LF oscillations of APD we found were also caused by sympathetic discharge. Yet, preliminary data of our group shows that stellectomy results in significant reduction in TdP inducibility in the CAVB dog, which implies that, even under anesthetic conditions, the sympathetic nervous system contributes for a great part to arrhythmogenesis in this model [abstract presented at AHA 2017 (van Weperen et al., 2017, unpublished data)]. Finally, it would have been of great interest to measure PRD in the CAVB dog, so we could evaluate if the intracardiac MAP oscillations correlate with PRD on the surface ECG. Unfortunately, PRD measurement requires transformation of the ECG to Frank leads with either Kors or inverse Dower’s matrix, which are not validated for the canine ECG. Furthermore, because of the complete AV-block, the T waves are often distorted by the interference of P waves, which impedes accurate measurement of beat-to-beat T wave changes.

## Conclusion

In the chronic AV block dog model, we observed oscillations of LV MAPD at respiratory frequency, which are augmented after remodeling compared to non-remodeled conditions. In addition, LF oscillations of MAPD difference were already altered acutely after creation of AV block and increased even further at chronic AV block conditions. Furthermore, CAVB dogs, that are susceptible to drug-induced TdP, show increased LF oscillations compared to their non-inducible counterparts.

## Data Availability

The datasets generated for this study are available on request to the corresponding author.

## Ethics Statement

The animal study was reviewed and approved by Centrale Commissie Dierproeven (Central Committee of Animal Experiments), The Hague, the Netherlands.

## Author Contributions

DS contributed in writing, data collection, and analysis. JB contributed in technical support. AB and AD conducted animal experiments. MV helped in supervision and review of manuscript.

### Conflict of Interest Statement

The authors declare that the research was conducted in the absence of any commercial or financial relationships that could be construed as a potential conflict of interest.
